# The effect of oxytocin, sublingual, and intrauterine misoprostol on blood loss in cesarean delivery: A randomized clinical trial

**DOI:** 10.1016/j.eurox.2025.100369

**Published:** 2025-02-03

**Authors:** Mahdieh Masoumzadeh, Vahideh Rahmani, Manizheh Sayyah-Melli, Anis Sani

**Affiliations:** a Women's Reproductive Health Research Center, Tabriz University of Medical Sciences, Tabriz, Iran; b Fellowship of Gynecologic Oncology. Women's Reproductive Health Research Centre, Tabriz University of Medical Sciences, Tabriz, Iran; c Tabriz University of Medical Sciences, Tabriz, Iran

**Keywords:** Misoprostol, Oxytocin, Postpartum hemorrhage, Cesarean Section

## Abstract

**Background:**

The efficacy of different uterotonic agents is yet to be determined.

**Methods:**

This was a randomized clinical trial on 240 pregnant mothers with a history of cesarean section in three groups: A: sublingual misoprostol and oxytocin, B: intrauterine misoprostol and oxytocin, and C: a higher dose of oxytocin alone. The intrapartum blood loss and the estimated blood loss within 24 h after surgery were compared between the groups.

**Results:**

The baseline characteristics showed no significant differences among the groups. The volume of blood loss during surgery and within 24 h postpartum did not differ significantly among the groups (A: 230.72 ± 97.30, B: 245.60 ± 88.50, C: 229.02 ± 109.78, p = 0.115, and A: 2023.84 ± 480.08, B: 2045.26 ± 598.99, C: 2025.61 ± 538.93, p = 0.819, respectively).

**Conclusion:**

Intrauterine misoprostol plus oxytocin, sublingual misoprostol plus oxytocin and a higher dose of oxytocin did not show any significant difference in the amount of blood loss during surgery and within 24 h post-operation.

**Clinical trial registration**

The trial was registered with the Iranian Registry of Clinical Trials (IRCT20100429003833N2) available at https://www.irct.ir/.

## Introduction

Intrapartum hemorrhage (IPH) and Postpartum hemorrhage (PPH) are the leading causes for maternal mortality, particularly in developing countries [1], [2] Complications caused by PPH can seriously threaten maternal health [3]. However, most deaths and complications caused by PPH are preventable [4].

In addition to the mechanical methods, various pharmacological agents have been used to control bleeding in cesarean delivery [5]. Oxytocin is the primary stimulant of uterine contractions and is frequently administered as prophylaxis to prevent uterine atony and excessive bleeding during cesarean delivery. Despite its beneficial effects, 10–40 % of individuals receiving oxytocin need additional uterotonic medication. [6]. In 2012, misoprostol was introduced as an alternative to oxytocin in WHO Recommendations for the Prevention and Treatment of Postpartum Hemorrhage [2]. Misoprostol, an analog of prostaglandin E1, demonstrates effective prevention and management of PPH due to its uterotonic properties. Misoprostol can be administered orally, sublingually, buccally, rectally, and by being placed within the uterus [7]. Few previous studies have investigated the efficacy of intrauterine misoprostol to decrease bleeding in cesarean delivery, however none of them compared it with sublingual misoprostol [8], [9], [10], [11], [12]. In a recent RCT, Alalfy et al. showed that intrauterine misoprostol combined with oxytocin is effective and safe in reducing PPH rate compared with oxytocin [10]. A Cochrane network meta-analysis of uterotonic drugs for managing PPH reported that the combination of misoprostol and oxytocin did not show a significant difference in the incidence of post-partum bleeding ≥ 1000 mL compared to oxytocin alone. However, the different routs of misoprostol administration were not compared. Moreover the adverse side effects of the uterotonic medications were often not discussed in the reviewed studies [13].

In this randomized clinical trial we compared the effects of sublingual misoprostol plus oxytocin, intrauterine misoprostol plus oxytocin, and oxytocin alone in controlling bleeding during cesarean delivery and 24 h after that.

## Materials and methods

This study was a prospective randomized clinical trial at Al-Zahra and Taleghani teaching hospitals in Tabriz, Iran, consistent with the Consolidated Standards of Reporting Trials (CONSORT) guidelines.

### Sample size calculation

A sample size of 159 participants was calculated using the G*Power tool with the effect size of 0.25 (Cohen's D) and the minimum type I and type II errors of 5 % and 20 %, respectively. We estimated a 0.25 standard deviation difference in mean intra-partum blood loss between the groups based on the difference of blood loss during caesarean section between intrauterine misoprostol and oxytocin in a previous research [8]. This small effect size was selected to ensure the study had sufficient power to detect even modest advantages of one intervention over the others [14]. Due to the multiple hypothesis tests and to prevent power reduction compared to the increase coefficient of 1.488, the final sample size was calculated as 237 participants and rounded up to 240 (80 in each group) [15].

### Participants and blinding

The study participants were pregnant women between the ages of 18 and 45 who had term pregnancies and a history of previous cesarean surgeries. These women were scheduled for cesarean deliveries and had provided informed consent to participate in the study. the exclusion criteria were multiple pregnancies, polyhydramnios (due to increased complications of atony and bleeding during childbirth), hemoglobin< 8 before surgery, records of allergy to prostaglandins, vaginal bleeding during pregnancy, coagulation disorders, and low platelets as well as preterm fetus, simultaneous uterine fibroids, total placenta previa and placenta accreta, a record of hypertension, heart disease, body mass index (BMI) ≥ 30, and need for additional uterotonics.

Randomization was conducted at a1:1:1 ratio using Excel software rand function. The allocation was performed by a blinded nurse using sealed envelopes. The sealed envelopes were delivered to the operating room (OR) when a participant enrolled in the study entered the OR. The surgical team members were unaware of the grouping before the time of intervention. The participants and the analyst were also blinded.

### Intervention

The intervention was applied after the placental removal, depending on the type of group. Group (A); this group received ten units of oxytocin (within 500 ccs of Ringer's solution) and 400 µg of sublingual misoprostol tablet (administered by anesthesiologist after removal of the placenta as a sublingual item with choking control). Group (B); this group received ten units of oxytocin (within 500 ccs of Ringer's solution) and 400 µg of intrauterine misoprostol (200 µg were placed in each cornua after removal of the placenta). Group (C); this group received 30 units of oxytocin (within 500 ccs of Ringer's solution). Also, 30 units of oxytocin were infused for all of the patients in the ward through one liter of solution for 8 h. The anesthesia method was spinal, and the caesarean delivery method was Pfannenstiel for the abdominal wall and low transverse for uterine incision.

### Outcome assessment

The primary outcome measure was intra-partum blood loss volume. The Secondary outcomes were the estimated early post-partum bleeding volume (24 h post-partum), the duration of surgery, and the rate of drugs side effects.

The amount of blood loss during surgery was assessed after removal of the placenta and until the end of uterine repair by measuring the volume of blood collected in the suction device and calculating the weight difference of abdominal gauze before and after surgery (one mL of blood was considered for each gram of weight difference).

The blood loss during delivery and the early postpartum period is roughly comparable to the volume of blood added during pregnancy, resulting in a stable hematocrit level in the acute stage and the first few days after delivery. However, if the hematocrit levels decrease after delivery, it indicates additional bleeding beyond the hypervolemia that occurs during pregnancy.

In this study, we estimated the volume of bleeding within 24 h post-partum by calculating the total volume increase during pregnancy, typically 30–60 % (1500–2000 mL) of the non-pregnant woman's blood volume due to normal and moderate hypervolemia, and then adding 500 mL for each 3 % decrease in hematocrit levels from the baseline [16]. It should be noted that this estimation is used for comparison purposes and is not a precise measure of the amount of blood loss.

The duration of the surgery was recorded from the time of skin incision to its complete repair.

The side effects of medicines, such as headache (using visual analogue scale), fever, shivering, nausea, and vomiting (N/V Score), were evaluated in all patients in the first 6 h after the surgery. In addition, the occurrence of uterine atony and the requirement for blood transfusion within the initial 24 h post-surgery were recorded.

### Statistical analysis

The data were analyzed using SPSS 26 software. The Kolmogorov-Smirnov test was used to determine the distribution of the data. For normally distributed data, a one-way ANOVA test was used for comparison, while non-normally distributed data were analyzed using The Kruskal-Wallis test. If a statistically significant result was found, pairwise comparison post hoc tests were used to determine the source of the difference. The chi-square test was performed to compare categorical variables. P-values less than 0.05 were considered statistically significant.

## Results

**Fig. 1:**
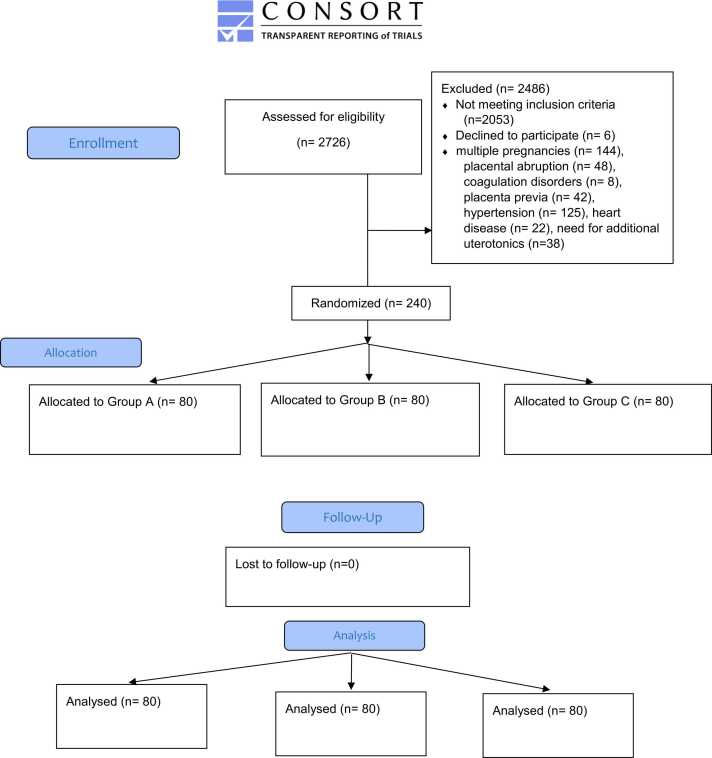
CONSORT Flow Diagram.

Two hundred-seventy eight patients were considered for enrollment; however, 38 participants were excluded from the study due to the need for more drug interventions to control the bleeding. Overall, this study included 240 participants who were selected and randomly divided into three groups (Fig. 1). All the included participants were followed up for 24 h after surgery and there was no missing data.

There was no statistically significant difference in the baseline characteristics between the groups (Table 1)**.**

**Table 1:** Basic characteristics of the study groups.

Groups	Median	IQR	Min-Max	P- value*
Age(Years)				
A	30	[26–75]	42–15	H(2)= 4.80 P = 0.90
B	32	[28. 25, - 36]	43–18	
C	31.5	[27–35]	42–18	
Gestational Age (Weeks)				
A	38	[37–39]	40–34	H(2)= 4.19 P = 0.123
B	38	[37–39]	40–34	
C	37	[29–41]	41–29	
BMI				
A	26.95	[23.88–28.89]	16.70–37.00	(H(2)= 5.16 P = 0.076
B	26.55	[24.72–28.87]	18.60–30.00	
C	25.90	[23.70–27.70]	16.20–30.00	
Newborn’s Weight(g)				
A	3300.00	[3194.02–3337.98]	4750–2110	H(2)= 11.61 P = 0.003
B	3200.00	[3056.67–3297.43]	4450–1450	
C	3090.00	[2854.04–3104.33]	3800–1400	
Baseline Hematocrit				
	Mean	CI_95 %_ For Mean		P-Value* *
A	36.04	[35.39–36.69]		F(2237)= 0.194 p = 0.824
B	36.34	[35.62–37.06]		
C	36.18	[35.50–36.87]		

* * Analysis of Variances was performed, and a p-value is reported.

* A Kruskal-Wallis H was performed, and a p-value was reported.

The IPH and early PPH volumes were not significantly different in the three groups (A: 230.72 ± 97.30, B: 245.60 ± 88.50, C: 229.02 ± 109.78, p = 0.115, and A: 2023.84 ± 480.08, B: 2045.26 ± 598.99, C: 2025.61 ± 538.93, p = 0.819, respectively) (Table 2). None of the participants needed blood transfusion 24 h post-delivery. Uterine atony was not seen in any of the patients.

**Table 2:** Blood loss volume and surgery duration.

Bleeding	Groups	N	Mean ± SD	†
During Surgery (mL)	A	80	230.72 ± 97.30	F(2237)= 0.627 P = 0.115
	B	80	245.60 ± 88.50	
	C	80	229.02 ± 109.78	
During 24 h After Surgery (mL)	A	80	2023.84 ± 480.08	F(2237)= 0.736 P = 0.819
	B	80	2045.26 ± 598.99	
	C	80	2025.61 ± 538.93	
Surgery duration (min)	A	80	57.31	F(2237)= 0.39 P = 0.094
	B	80	53.56	
	C	80	56.38	

†one-way ANOVA was performed to test for differences between groups A,B and C.

The surgery duration in the intrauterine misoprostol recipient group was significantly lower than the sublingual misoprostol recipient group (A: 57.31 min, B: 53.56 min, H(2)= 2.88, p = 0.012). However, this difference was not clinically significant. At the same time, the analysis of the surgery duration results in other groups was not significantly different from each other (Table 2)

The side effects of medications, such as fever, nausea, emesis, and headache were recorded within 6 h postpartum, and none of the above-stated side effects were observed in any of the patients. Nevertheless, the incidence of postpartum shivering was greater in group A when compared to groups B and C (A: 74.51 %, B: 9.80 %, C: 15.69 %, p < 0.001) (Table 3).

**Table 3:** Side effects rate.

Groups	Shivering n (%)[AR* ]	Χ(df),p-value* *
A	38 (%74.51)[0,7]	χ(2) = 49.74, P < 0.001
B	5 (%9.80)[−4,0]	
C	8 (%15.69)[−3,0]	

* * Chi-square test of independence with effect sizes and corresponding p-value is reported

## Discussion

This was a randomized clinical trial comparing three interventions for controlling intrapartum and early postpartum bleeding in cesarean delivery: 400 μg sublingual misoprostol plus 10 units of oxytocin, 400 μg intrauterine misoprostol plus 10 units of oxytocin, and 30 units of oxytocin alone. Our findings showed that the volume of blood loss was not significantly different between the groups, however the surgery duration in the intrauterine misoprostol group was lower. Participants did not experience any medication side effects except for shivering in the sublingual misoprostol recipients.

In this study we tested the intrauterine administration of misoprostol. Some beneficial features of intrauterine administration are the ease of administration, especially during cesarean delivery, and the possibility to administer in both general and spinal anaesthesia without contaminating the surgical field compared to rectal route. According to the mechanism of misoprostol, i.e. binding to myometrial cells and creating powerful contractions starting from the fundus near the uterine horn (cornua), it seemed that the progression of these contractions downwards from the fundus towards the internal os to remove the tissue and reduce bleeding may possibly be achieved faster and better with direct placement of this medicine in the cornea, which is the beginning place of uterine contraction rhythms [12].

According to the results of this study, the bleeding volume during surgery and 24 h post-operation were not significantly different among three groups. In a study by Tiwari et al., which aimed at comparing intrauterine misoprostol (400 μg) and oxytocin (20 units) with oxytocin (20 units) alone, the blood loss volume in the misoprostol recipients was significantly less than the oxytocin recipients [17]. In another trial by Rasri et al., the mean hemorrhage volume after cesarean delivery in the oxytocin (20 units) and misoprostol (400 μg per corner of the uterus) recipient group was 35.36 % less than the oxytocin recipient group. Alalfy et al., showed that when intrauterine misoprostol (400 μg) plus oxytocin (10 units) was compared with oxytocin alone (10 units), the blood loss volume in the misoprostol recipients was 25.62 % lower than the control group [10]. Also, in a study by Bahadur et al., comparing intrauterine misoprostol (800 μg) plus oxytocin with intravenous oxytocin alone, the hemorrhage volume in the misoprostol recipient group was lower (p = 0.080) and none of the patients required a higher dose of oxytocin [8]. In all the above-stated studies, the hemorrhage volume in the misoprostol and oxytocin recipient group was significantly lower than in those receiving oxytocin alone. In these studies, the control and the intervention groups received misoprostol as the second uterotonic agent for controlling bleeding. In this study, the group C received 20 more units of oxytocin, and the other two groups received misoprostol to check and compare the effect of the drug. Hence, the comparison of the effect of additional misoprostol with oxytocin alone was evaluated in a way not performed previously. It appears that 400 μg intrauterine or sublingual misoprostol acts similarly to 20 units of oxytocin in controlling hemorrhage in this study.

This study also compared intrauterine and sublingual misoprostol and found no significant difference in blood loss volume between the two methods. Therefore, if sublingual administration is not feasible due to general anesthesia or nausea, the intrauterine form can be used with similar results. In a study by Moutaz M et al. with the objective of comparing the efficacy of rectal misoprostol (400 μg) with intrauterine misoprostol (200 μg per each corner of the cornua), the hemorrhage volume in both groups was not significantly different (p = 0.979) [18].

The shivering side effect in the sublingual misoprostol recipient group was significantly higher than the other two groups in our study, and other side effects, including headache, nausea/vomiting, and fever, were not observed in any of the groups.

### Clinical and research implications

Our study suggests that intrauterine misoprostol can effectively complement oxytocin for hemorrhage control during cesarean delivery, yielding outcomes similar to an additional 20 units of oxytocin while causing fewer adverse effects like shivering compared to the sublingual route. Further research is needed to establish the optimal dosage and administration route for misoprostol. Comparing misoprostol alone with oxytocin could yield clearer results, ensuring patient safety. Future studies should also evaluate the cost-effectiveness of misoprostol use at the surgeon's discretion versus routine prevention and explore its application in high-risk pregnancies.

### Strengths and limitations

This study is one of the few randomized clinical trials comparing intrauterine and sublingual misoprostol with oxytocin in hemorrhage prevention in cesarean delivery. Another strength of this study was the precise measurement of intra-operative blood loss. However, the post-partum blood loss volume was based on an estimation and was used only for comparative purposes. Limitations include the exclusion of high-risk and obese women, affecting generalizability, and hemoglobin measurement at 24 h instead of the optimal 48. Nonetheless, the findings may support future meta-analyses for stronger recommendations on hemorrhage prophylaxis in cesarean deliveries.

## Conclusion

There was no significant difference in the amount of blood loss during the cesarean delivery and 24 h post-operation among recipients of intrauterine misoprostol, sublingual misoprostol, and a higher dose of oxytocin. However, the duration of surgery with intrauterine administration is shorter, and the rate of shivering complication is lower than in the sublingual form.

## Ethical standards

The study adhered to the principles of the Declaration of Helsinki.

## Ethical approval

The ethics committee of Tabriz University of Medical Sciences reviewed and approved this study (IR.TBZMED.REC.1400.409).

## Author Contributor

M M, V R, and M S contributed to study design, data collection, and article writing, and A S contributed to article writing and revising it.

## Funding

This study was supported by the Women's Reproductive Health Research Centre, Tabriz University of Medical Sciences.

## CRediT authorship contribution statement

**Anis Sani:** Writing – review & editing, Writing – original draft. **Sayyah-Melli Manizheh:** Supervision, Project administration, Conceptualization. **Rahmani Vahideh:** Supervision, Project administration, Methodology. **Masoumzadeh Mahdieh:** Writing – original draft, Project administration, Methodology, Investigation, Conceptualization.

## Declaration of Competing Interest

The authors report no conflict of interest.
